# Impact of Geographic Location on Vitamin D Status and Bone Mineral Density

**DOI:** 10.3390/ijerph13020184

**Published:** 2016-02-02

**Authors:** Kyung-Jin Yeum, Byeng Chun Song, Nam-Seok Joo

**Affiliations:** 1 Division of Food Bioscience, College of Biomedical and Health Sciences, Konkuk University, Chungju-si 27478, Korea; kyeum@kku.ac.kr (K.-J.Y.); bcsong@kku.ac.kr (B.C.S.); 2 Department of Family Practice and Community Health, Ajou University School of Medicine, Suwon-si 16499, Korea

**Keywords:** bone mineral density, vitamin D status, geographic location, serum 25-hydroxyvitamin D

## Abstract

A significant decline of serum 25-hydroxyvitamin D concentration [25(OH)D] with increasing latitude has been reported only for Caucasians. To determine the association between serum 25(OH)D and geographic location and its impact on bone mineral density (BMD) in an Asian population, a total of 17,508 subjects (8910 men and 8598 women) from the 2008–2010 Korea National Health and Nutrition Examination Survey (KNHANES) were stratified into four age groups and analyzed for 25(OH)D and BMD according to geographic location (South, 33° N–35° N; Middle, 36° N; North, 37° N–38° N). Mean 25(OH)D were 47.7 and 41.2 nmol/L; calcium intake, 564.9 & 442.3 mg/d; femoral neck BMD, 0.829 & 0.721 g/cm^2^; and lumbar spine BMD, 0.960 & 0.918 g/cm^2^ for men and women, respectively. Both men and women living in the South had significantly higher 25(OH)D and femoral neck BMD for those ≥50 years old. Lumbar spine BMD was significantly higher in men ≥50 years old, and for women 10–29 & 50–69 years old living in the South. A 1 or 2 degree difference in latitude has a significant effect on serum 25(OH)D and BMD in this low vitamin D status population. Thus, consideration of geographic location for a recommendation of vitamin D intake may be necessary.

## 1. Introduction

The skin production of vitamin D_3_ from 7-dehydrocholesterol by ultraviolet B (UVB) exposure is well known [1]. The photosynthesis of vitamin D_3_ can be particularly important for a population with inadequate vitamin D status. Widespread vitamin D deficiency and insufficiency has been reported in many different areas of Asian countries including China [2], India [3], Japan [4] and Korea [5]. Considering limited food sources for vitamin D such as fatty fish [6], solar exposure can be one of the major factors contributing to vitamin D status in these populations. Undoubtedly, the role of vitamin D for bone health [7] can be pivotal. Although the Institute of Medicine (IOM) report indicated no beneficial effect of a serum 25-hydroxyvitamin D [25(OH)D] concentration above 50 nmol/L [8], their findings may not be relevant for these populations. In addition, calcium deficiency in Asians such as Chinese [9], Indians [10], Japanese [4], and Koreans [11,12,13] emphasizes the importance of adequate vitamin D status for proper bone mass development and maintenance.

The importance of the angle of sunlight splitting 7-dehydrocholesterol in the skin is well documented [1,14,15]. Notably, no vitamin D was reported to be synthesized between November and March in Boston (42° N) for residents [14], and vitamin D_3_ production in the skin is reported to be minimal in winter at latitudes above 35° [1]. In another study, it has been reported that solar exposure of 3.5 and 22.4 min in July and winter, respectively in Tsukuba (36° N), and 76.4 min in winter in Sapporo (43° N), were required for synthesis of 5.5 µg vitamin D_3_ production in the face and the back of hands under a cloudless sky at noon. Furthermore, extended solar exposure time of 106 min and 271 min were estimated to be required at 9:00 a.m. and 3:00 p.m. in the same region [16] to synthesize the same amount. Nevertheless, an ecologic meta-regression analysis indicated that there was no overall influence of latitude for vitamin D status in Caucasians with lighter skin as well as non-Caucasians with darker skin [17]. In this study, the mean serum 25(OH)D concentration was 54 nmol/L, which is considerably higher than the vitamin D status in many Asian countries. The impact of geographic location on vitamin D status and bone mass in an Asian population with low vitamin D status is unknown. Thus, it is important to understand the contributions of geographic location to vitamin D status and bone mass in population with widespread vitamin D deficiency in order to target interventions most effectively. Therefore, we aimed to evaluate the importance of geographic location on vitamin D status and bone mineral density using data from the fourth and fifth Korea National Health and Nutrition Examination Survey (KNHANES).

## 2. Methods and Materials

### 2.1. Study Population

**Figure 1:**
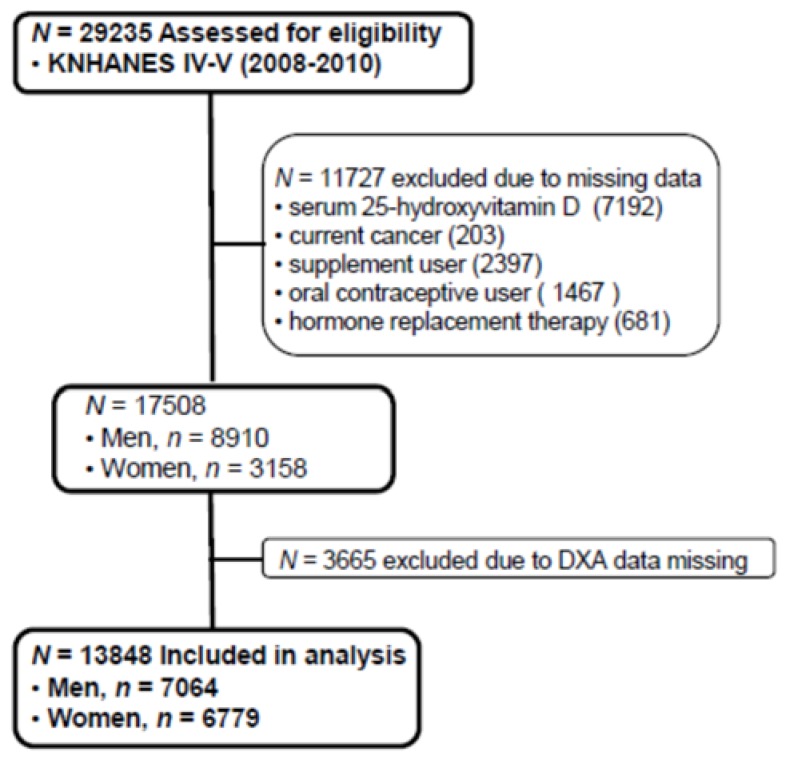
Flow diagram of subject inclusion and exclusion in the Korea National Health and Nutrition Examination Surveys (KNHANES IV & V). DXA: Dual energy X-ray absorptiometry.

The Korean National Health and Nutrition Examination Survey (KNHANES), conducted periodically by the Korea Centers for Disease Control and Prevention since 1998, provides comprehensive information on health status, health behavior, nutritional status, and socio-demographics in national districts in Korea. The fourth (IV-2 and IV-3, 2008, 2009) and fifth (V-1, 2010) KNHANES data containing serum 25(OH)D concentration and Dual X-ray Energy Absorptiometry (DXA) were used in this cross-sectional analysis. From an initial total of 29,235 males and females, 11,727 subjects (missing data of serum 25(OH)D, current cancers, any supplement user, oral contraceptive users, hormone replacement therapy) were excluded. Of 17,508 evaluated subjects, 3665 subjects were also excluded for missing data of DXA. A final 13,843 participants (7064 males and 6779 females) were used in this analysis as shown in Figure 1. All participants provided written informed consent before taking the survey.

### 2.2. Laboratory and Nutritional Assessment

Blood samples were collected year-round after an 8-h fasting. Samples were immediately processed, refrigerated, and transported via cold storage to the central testing institute (NeoDin Medical Institute, Seoul, South Korea) where they were then analyzed within 24 h. Serum 25(OH)D concentration was measured with a radioimmunoassay (RIA) kit (DiaSorin Inc., Stillwater, MN, USA) using an r-counter (1470Wizard; PerkinElmer, Turku, Finland). The inter-assay coefficients of variation (CV) were 2.8%–6.2% for samples in 2008–2009 and 1.9%–6.1% for samples in 2010. Serum 25(OH)D was measured in the same institute, which conducted quality control measures every other week throughout the analysis period in order to minimize analytical variation. Bone mineral density (BMD) (g/cm^2^) of the lumbar spine (L1–4) and femoral neck was measured by dual-energy X-ray absorptiometry (DISCOVERY-W fan-beam densitometer, Hologic Inc., Bedford, MA, USA) with CVs of 1.9% and 2.5%, respectively. Nutrient intakes, including total calorie and calcium intakes, were assessed with a 24-h dietary recall questionnaire administered by a trained dietician. The results were calculated using the Food Composition Table developed by the National Rural Resources Development Institute (7th revision).

### 2.3. Lifestyle Questionnaires

Physical activity was assessed by a questionnaire and categorized as “yes” or “no”; “yes” meaning >30 min of moderate physical activity for three or more times in the last week. Current smokers were defined as those who were currently smoking and had smoked more than five packs of cigarettes during their life; ex-smokers were persons who had smoked in the past but had quit; nonsmokers had no history of smoking. Regular alcohol drinkers were those who drank alcohol more than one time per month, and nondrinkers were all others. Occupations were classified into two groups: indoor workers and outdoor workers. Indoor workers included managers, professionals and related workers, clerical office workers, service workers, and sales workers. Unemployed subjects were classified as indoor workers considering their limited outdoor activities. Outdoor workers included skilled agricultural, forestry, and fishery workers.

### 2.4. Statistical Analysis

The Korea Centers for Disease Control and Prevention recommended using the KNHANES data by complex sample analysis through proper data weighting. The data of males and females were divided into age groups (10 to 29, 30 to 49, 50 to 69, and >70-years-old) and the latitude in which they lived in. The latitudes of South Korea ranges from 33° to 38° and the KNHANES data were composed of city unit. Therefore, we divided geographical locations into three different regions, South (33° to 35°), Middle (36°) and North (37° to 38°). General characteristics including age, body weight, body mass index (BMI), fat mass (measured by Dual Energy X-ray Absorptiometry), daily calcium intakes, serum 25(OH)D, femur neck, and lumbar spine BMD were evaluated after weighing all values without any adjustment using a general linear model. In addition, smoking, drinking, and moderate physical activity was evaluated by a χ^2^ test in both genders. We compared serum 25(OH)D concentration and BMD (total hip, femur neck, and lumbar spine) according to each age group (10 to 29, 30 to 49, 50 to 69, and >70-years-old) and region (South, Middle, North) by ANCOVA test after adjustments for variables affecting serum 25(OH)D concentrations. These variables were total body fat, smoking status, alcohol intake, moderate activity and education for men; total body fat, smoking status, alcohol intake, moderate activity, education, menopause, oral contraceptive use, and hormone replacement therapy for women. All *p*-values were *p* for trend to assess the significance of all analyses and *p* < 0.05 was considered significant. Data were analyzed using SPSS 19.0 (SPSS Inc., Chicago, IL, USA).

## 3. Results

### 3.1. General Characteristics of Study Subjects Categorized by Region and Age

General characteristics of men and women by regions are presented in Table 1. Mean calcium intakes for men were 570.8, 550.8 and 567.9 mg/d, and for women were 448.0, 400.3 and 448.7 mg/d in the North, Middle and South, respectively. Mean serum 25(OH)D for men were 46.0, 52.7 and 51.1 nmol/L (*p* < 0.001) in the North, Middle and South. Male fat mass was highest in the North (16.1 kg), followed by the Middle (15.5 kg), and the lowest in the South (14.8 kg). The mean serum 25(OH)D for women were 39.7, 44.1 and 44.6 nmol/L (*p* < 0.001) in the North, Middle and South, respectively. There was no significant difference in fat mass with respect to regions in women (18.9, 18.8 and 18.4 kg for the North, Middle and South). The serum 25(OH)D sampling was evenly distributed across the four seasons since KNHANES was conducted year-round. Men living in the North, Middle and South had mean femoral neck bone mineral density (BMD) of 0.834, 0.808 and 0.827 g/cm^2^, respectively, and Lumbar spine BMD of 0.959, 0.949 and 0.963 g/cm^2^, respectively. Women living in the North, Middle and South presented mean femoral neck bone mineral density (BMD) of 0.725, 0.706 and 0.720 g/cm^2^, respectively, and Lumbar spine BMD of 0.922, 0.892 and 0.918 g/cm^2^, respectively. Almost half of the subjects were living in the North (48.5% and 46.9% men and women, respectively), followed by the South (39.8% and 40.9% men and women, respectively), with the smallest number of subjects living in the Middle (11.7% and 12.2% men and women, respectively).

**Table 1:** General characteristics of study subjects by regions and genders.

**Men (*n* = 7064)**				
	**North (37° N)**	**Middle (36° N)**	**South (33° N~36° N)**	***p***
Numbers, *n* (%)	3428 (48.5)	824 (11.7)	2811 (39.8)	
Age (years)	39.9 (0.4)	44.0 (1.0)	41.3 (0.6)	<0.001
Body weight (kg)	69.9 (0.3)	69.0 (0.7)	68.0 (0.3)	<0.001
BMI (kg/m^2^)	24.0 (0.1)	24.0 (0.2)	23.5 (0.1)	<0.001
Fat mass (kg)	16.1 (0.2)	15.5 (0.3)	14.8 (0.2)	<0.001
Calcium intake (mg/d)	570.8 (8.3)	550.8 (18.9)	567.9 (9.5)	0.629
25OHD (nmol/L)	46.0 (0.7)	52.7 (1.7)	51.1 (0.9)	<0.001
FNBMD (g/cm^2^)	0.834 (0.003)	0.808 (0.007)	0.827 (0.004)	0.004
LSBMD (g/cm^2^)	0.959 (0.003)	0.949 (0.007)	0.963 (0.004)	0.244
Smoking *, *n* (% ^†^)				
Current	1336 (44.1)	312 (41.5)	1156 (45.6)	<0.001
Past and never	1696 (55.9)	440 (58.5)	1380 (54.4)	
Alcohol intake *, *n* (% ^†^)				
Yes	2282 (75.7)	552 (73.5)	1887 (74.4)	<0.001
No	733 (24.3)	199 (26.5)	648 (25.6)	
Physical activity *, *n* (% ^†^)				
Yes	329 (10.9)	146 (19.4)	375 (14.8)	<0.001
No	2701 (89.1)	606 (80.6)	2160 (85.2)	
Occupation *, *n* (% ^†^)				
Indoor workers	2904 (84.7)	687 (83.4)	2423 (86.2)	<0.001
Outdoor workers	243 (7.1)	87 (10.6)	192 (6.8)	
No jobs	282 (8.2)	50 (6.1)	196 (7.0)	
**Women (*n* = 6779)**				
	**North (37° N)**	**Middle (36° N)**	**South (33° N~36° N)**	***p***
Numbers, *n* (%)	3182 (46.9)	826 (12.2)	2771 (40.9)	
Age (years)	37.0 (0.4)	42.6 (1.5)	42.5 (0.7)	0.001
Body weight (kg)	56.5 (0.2)	56.4 (0.3)	56.5 (0.2)	0.975
BMI (kg/m^2^)	22.7 (0.1)	23.0 (0.2)	22.9 (0.1)	0.173
Fat mass (kg)	18.9 (0.2)	18.8 (0.3)	18.4 (0.2)	0.079
Calcium intake (mg/d)	448.0 (6.7)	400.3 (10.4)	448.7 (8.9)	<0.001
25OHD (nmol/L)	39.7 (0.6)	44.1 (1.5)	44.6 (0.8)	<0.001
FNBMD (g/cm^2^)	0.725 (0.003)	0.706 (0.009)	0.720 (0.004)	0.132
LSBMD (g/cm^2^)	0.922 (0.004)	0.892 (0.010)	0.918 (0.004)	0.029
Smoking *, *n* (% ^†^)				
Current	196 (6.8)	43 (5.8)	108 (4.3)	<0.001
Past and never	2668 (93.2)	695 (94.2)	2420 (95.7)	
Alcohol intake *, *n* (% ^†^)				
Yes	1178 (41.4)	261 (35.4)	945 (37.4)	<0.001
No	1670 (58.6)	477 (64.6)	1583 (62.6)	
Physical activity *, *n* (% ^†^)				
Yes	254 (8.9)	178 (24.2)	384 (15.2)	<0.001
No	2610 (91.1)	559 (75.8)	2144 (84.8)	
Occupation *, *n* (% ^†^)				
Indoor workers	2737 (86.0)	695 (84.1)	2398 (86.5)	<0.001
Outdoor workers	252 (7.9)	79 (9.6)	208 (7.5)	
No jobs	193 (6.1)	52 (6.3)	165 (6.0)	

Data represent mean (SE, standard error) after weighing in complex sample analysis without adjustment. BMI: body mass index, Fat mass was measured by Dual-energy-X ray Absorptiometry (DXA); 25OHD: serum 25-hydroxyvitamin D concentration; FNBMD: femur neck bone mineral density; LSBMD: lumbar spine bone mineral density; * missing data were not included; ^†^ percents of total study subjects in each region.

Table 2 presents the general characteristics of study subjects categorized by the four age groups. For both men and women, the lowest serum 25(OH)D concentration was in the 10 to 29-year-old age group (42.7 and 38.9 nmol/L). On the other hand, calcium intake was the lowest in the age group ≥70-year-old for both men (453.5 mg/d) and women (327 mg/d). 

**Table 2:** General characteristics of study subjects categorized by age.

**Age Groups of Men (*n* = 7064)**					
	**10~29 (*n* = 1574)**	**30~49 (*n* = 2548)**	**50~69 (*n* = 2164)**	**≥70 (*n* = 778)**	***p***
Age (years)	20.0 (0.2)	39.6 (0.2)	57.7 (0.2)	75.2 (0.2)	
Body weight (kg)	66.1 (0.4)	71.9 (0.3)	67.7 (0.3)	61.4 (0.4)	<0.001
BMI (kg/m^2^)	22.5 (0.1)	24.4 (0.1)	24.1 (0.1)	22.8 (0.1)	<0.001
Fat mass (kg)	15.1 (0.2)	16.2 (0.2)	15.2 (0.2)	14.3 (0.3)	<0.001
Calcium intake (mg/d)	539.8 (10.0)	592.0 (8.0)	579.1 (9.2)	453.5 (14.5)	<0.001
25OHD (nmol/L)	42.7 (0.5)	47.9 (0.5)	53.2 (0.7)	52.4 (1.0)	<0.001
Physical activity, *n* (%)	123 (14.4)	331 (13.0)	293 (13.6)	103 (13.4)	0.162
FNBMD (g/cm^2^)	0.887 (0.005)	0.840 (0.003)	0.776 (0.003)	0.674 (0.005)	<0.001
LSBMD (g/cm^2^)	0.939 (0.006)	0.983 (0.003)	0.955 (0.004)	0.915 (0.008)	<0.001
Geography *, *n* (%)					
North (37° N)	832 (52.9)	1296 (50.9)	976 (45.1)	325 (41.8)	
Middle (36° N)	151 (9.6)	267 (10.5)	286 (13.2)	120 (15.4)	
South (33° N~35° N)	591 (37.5)	985 (38.6)	902 (41.7)	333 (42.8)	
**Age Groups of Women (*n* = 6779)**					
	**10~29 (*n* = 1554)**	**30~49 (*n* = 2630)**	**50~69 (*n* = 1747)**	**≥70 (*n* = 848)**	***p***
Age (years)	19.7 (0.2)	39.6 (0.1)	57.8 (0.2)	76.1 (0.2)	
Body weight (kg)	53.4 (0.3)	58.0 (0.2)	58.2 (0.2)	53.3 (0.4)	<0.001
BMI (kg/m^2^)	20.9 (0.1)	23.0 (0.1)	24.5 (0.1)	24.0 (0.1)	<0.001
Fat mass (kg)	17.5 (0.2)	18.9 (0.1)	20.1 (0.2)	18.4 (0.3)	<0.001
Calcium intake (mg/d)	438.7 (8.2)	469.2 (6.3)	449.2 (9.1)	327.0 (11.5)	<0.001
25OHD (nmol/L)	38.9 (0.5)	40.9 (0.5)	44.9 (0.5)	44.2 (0.7)	<0.001
Physical activity, *n* (%)	92 (9.8)	325 (12.4)	304 (17.6)	95 (11.3)	<0.001
FNBMD (g/cm^2^)	0.763 (0.004)	0.761 (0.003)	0.665 (0.003)	0.531 (0.004)	<0.001
LSBMD (g/cm^2^)	0.917 (0.004)	0.996 (0.003)	0.843 (0.004)	0.730 (0.006)	<0.001
Geography *, *n* (%)					
North (37° N)	806 (51.9)	1358 (51.6)	714 (40.9)	304 (35.8)	
Middle (36° N)	192 (12.3)	255 (9.7)	248 (14.2)	131 (15.5)	
South (33° N~36° N)	556 (35.8)	1017 (38.7)	785 (44.9)	413 (48.7)	

Data represent mean (SE). *p* values are *p* for trend from General linear model after data weighing in complex sample analysis without adjustment. BMI: body mass index; WC: waist circumference; Fat mass was measured by Dual-energy-X ray Absorptiometry (DXA); FNBMD: femur neck bone mineral density; LSBMD: lumbar spine bone mineral density. Physical activity: >30 min of moderate physical activity for three or more times in the last week. * *p* values were from χ^2^ test.

Mean fat mass and BMI were highest in men of 30 to 49-year-old (16.2 kg and 24.4 kg/m^2^) and women 50 to 69-year-old (20.1 kg and 24.5 kg/m^2^) age groups. The femoral neck BMD was the highest in the 10 to 29-year-old age group in both men and women. With each progressively older age group, the femoral neck BMD of men declined to 94%, 87.5% and 76% at 30 to 49, 50 to 69 and ≥70-year-old age groups, respectively, and 99.7%, 87.2% and 70% at 30 to 49, 50 to 69 and ≥70-year-old age women, respectively; as compared to the 10 to 29-year-old age group. Both men and women had the highest lumbar spine BMD in the 30 to 49-year-old age group. The men in the ≥70-year-old age group had the lumber spine BMD over 93% compared to the 30 to 49-year-old age group men whereas women in the ≥70-year-old age group declined to 73.3% compared to the 30 to 49-year-old age group women.

### 3.2. Serum 25(OH)D Concentrations Categorized by Geographic Location and Age

**Figure 2:**
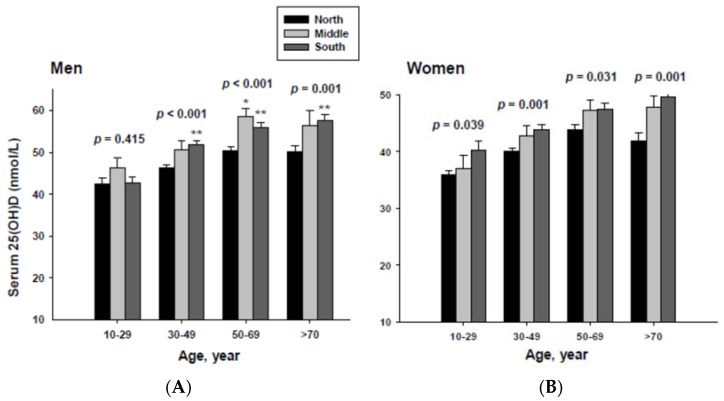
Adjusted mean serum 25(OH)D concentrations according to age and geographic location in men (**A**) and women (**B**). The *p* values are *p* for trend in the same group from ANCOVA test adjusted for total body fat, smoking, job, alcohol intake, moderate activity and education for men, and for total body fat, smoking, job, alcohol intake, moderate activity, education, menopause, oral contraceptive use and hormone replacement therapy for women Subject numbers for men are 10–29 years old, 832, 151, 591; 30–49 years old, 1296, 267, 985; 50–69 years old, 976, 286, 902; ≥70 years old, 325, 120, 333 for North, Middle and South, respectively, and for women are 10–29 years old, 806, 192, 556; 30–49 years old, 1358, 255, 1017; 50–69 years old, 714, 248, 785; ≥70 years old, 304, 131, 413 for North, Middle and South, respectively. * *p* < 0.05, North *vs.* Middle; ** *p* < 0.05 North *vs.* South.

The impact of geographic location on serum 25(OH)D concentration was examined by age group. The serum 25(OH)D concentrations were adjusted with potential confounders. The adjusted mean serum 25(OH)D concentrations by age and geographic location are shown in Figure 2. The serum 25(OH)D concentration in both men and women living in the South were significantly higher than those living in the North in all age groups except for men in the 10 to 29 year-old age group.

### 3.3. Bone Mineral Density Categorized by Geographic Location and Age

**Figure 3:**
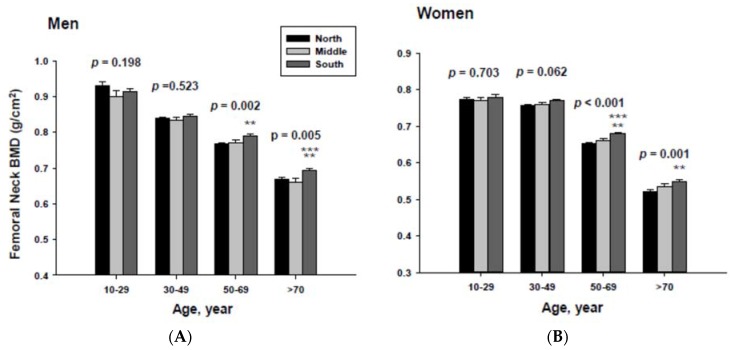
Adjusted mean femoral neck bone mineral densities according to age and geographic location in men (**A**) and women (**B**). The *p* values are *p* for trend in the same group from ANCOVA test adjusted for total body fat, smoking, job, alcohol intake, moderate activity and education for men, and for total body fat, smoking, job, alcohol intake, moderate activity, education, menopause, oral contraceptive use and hormone replacement therapy for women. Subject numbers for men are 10–29 years old, 832, 151, 591; 30–49 years old, 1296, 267, 985; 50–69 years old, 976, 286, 902; ≥70 years old, 325, 120, 333 for North, Middle and South, respectively, and for women are 10–29 years old, 806, 192, 556; 30–49 years old, 1358, 255, 1017; 50–69 years old, 714, 248, 785; ≥70 years old, 304, 131, 413 for North, Middle and South, respectively.** *p* < 0.05, North *vs.* South; *** *p* < 0.05 Middle *vs.* South.

The impact of geographic location on bone mineral density was examined by age category after proper adjustment. The adjusted mean femoral neck BMD are shown in Figure 3. The femoral neck BMD for people living in the South was significantly higher than those of men and women living in the North at the 50 to 69 and ≥70-year-old age groups. 

**Figure 4:**
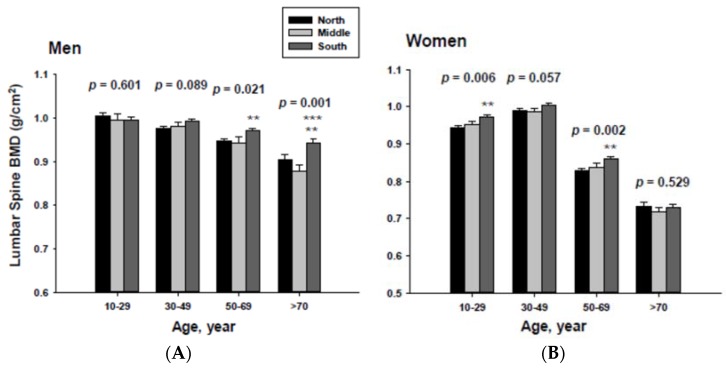
Adjusted lumbar spine bone mineral densities according to age and geographic location in men (**A**) and women (**B**).The *p* values are *p* for trend in the same group from ANCOVA test adjusted for total body fat, smoking, job, alcohol intake, moderate activity and education for men, and for total body fat, smoking, job, alcohol intake, moderate activity, education, menopause, oral contraceptive use and hormone replacement therapy for women. Subject numbers for men are 10–29 years old, 832, 151, 591; 30–49 years old, 1296, 267, 985; 50–69 years old, 976, 286, 902; ≥70 years old, 325, 120, 333 for North, Middle and South, respectively, and for women are 10–29 years old, 806, 192, 556; 30–49 years old, 1358, 255, 1017; 50–69 years old, 714, 248, 785; ≥70 years old, 304, 131, 413 for North, Middle and South, respectively. ** *p* < 0.05, North *vs.* South; *** *p* < 0.05 Middle *vs.* South.

In addition, men living in the South had significantly higher femoral neck BMD than those men living in the Middle at the ≥70-year-old age. Women living in the south also had significantly higher femoral neck BMD than those women living in the Middle at the 60 to 69-yeal-old age group. As presented in Figure 4, those men living in the South had significantly higher lumbar spine BMD than those living in the North at the 50 to 69 and ≥70-year-old age men groups as well as those living in the Middle at the ≥70-year-old age men group. Those women living in the South also had significantly higher lumbar spine BMD than those living in the North at the 10 to 29 and 50 to 69-year-old age women groups.

## 4. Discussion

This study examined serum 25(OH)D concentration and BMD in relation to geographic location in a population with a high prevalence of low serum 25(OH)D and calcium intake. Due to the nature of this study, this cross-sectional study cannot establish causality. However, it does confirm that serum 25(OH)D concentration as well as BMD with a 1–2 degree difference in latitude can have a significant effect on vitamin D status. Although skin production of vitamin D_3_ by solar exposure is well established [1,16], an ecologic meta-regression analysis reported no overall influence of latitude for vitamin D status [17]. In contrast, our study identified significant association between geographic location and vitamin D status. Furthermore, significant associations between geographic location and BMD were also found in our study. Unlike previous meta-analysis conducted in subjects with relatively adequate vitamin D status with mean serum 25(OH)D concentration of 54 nmol/L, the current study population in Korea had mean serum 25(OH)D concentrations of 47.7 and 41.2 nmol/L for men and women, respectively. Notably, latitude has been reported to be a strong determinant of the relative contribution of different behavioral factors analyzed by a multiple regression model [18]. In addition, a recent study [19] indicated that location was one of the strongest factors associated with serum 25(OH)D concentrations <75 nmol/L in subjects with human immunodeficiency virus. These findings emphasize that geographic location can be an important factor for vitamin D status in a population with vitamin D insufficiency.

Our findings indicate that subjects living in the South (33° N to 35° N) had significantly higher serum 25(OH)D concentration as well as higher BMD of the femoral neck and lumbar spine than those living in the North (37° N to 38° N). This implies that about 40% of the study population living in the North would benefit from increasing the duration of solar exposure as presented previously [16]. The current study subjects were divided into three different regions, South (33° N–35° N), Middle (36° N) and North (37° N to 38° N), with consideration to minimal vitamin D_3_ production in the skin during the winter at latitudes above 35° [1] as well as Asians’ higher threshold for skin production of vitamin D compared to that of Caucasians [20]. This study indicates that there was no significant difference of serum 25(OH)D concentration according to geographic location for residents living above 35° N. It is possible that there was minimal vitamin D_3_ production during the winter in the subjects living in the Middle and North. However, due to the small number of subjects living in the Middle, it is likely that this cohort does not have enough statistical power to show any significant difference.

It has been reported that ethnicity is a powerful modifier of the rate of BMD loss although body weight can also be a major determinant [21]. The annual rate of bone loss during the late perimenopause and the early post-menopause in the spine has been reported to be 1.8%–2.3% resulting in 7%–10% loss for 5 years [21]. As reported previously, Asian women had the most rapid BMD loss followed by Caucasian women, with the slowest BMD loss in African-American women. BMD loss of our study subjects were markedly accelerated during a menopause period resulting in ~30% difference in femoral neck and lumber spine BMD among different age groups in women. Although we do not know the optimal values of serum 25(OH)D and calcium intake for maintaining BMD in this population, it is clear that vitamin D status, which has been affected by the geographic location, plays a large role on bone loss.

Various factors are attributed to serum 25(OH)D concentrations such as skin synthesis of vitamin D_3_, intake of vitamin D-rich foods, and vitamin D-fortified foods. Considering the low intake of vitamin D-rich foods [22] and limited access to vitamin D-fortified foods [23] in this vitamin D insufficient population, this lends additional weight to the possibility of the potential impact of increased solar exposure on vitamin D status and consequent bone health. We were unable to assess the regional differences stratified by season due to the limitation of individual data access for both the season and region.

## 5. Conclusions

In conclusion, geographic location is a significant determinant of serum 25(OH)D concentration and BMD in this population with a high prevalence of low vitamin D and calcium status. This cross-sectional study suggests that a population living in any geographic location higher than 35° N may need to consider increasing of solar exposure or vitamin D intake. In contrast to previous findings in populations with an adequate vitamin D status, geographic location does impact overall vitamin D status and consequently bone health in this vitamin D insufficient population. A majority of the population in Korea living in a geographic location higher than 35° N are recommended to further increase solar exposure and/or vitamin D intake for improving bone mass in this at-risk population.
